# Performance characterization of siemens primus linear accelerator under small monitor unit and small segments for the implementation of step-and-shoot intensity-modulated radiotherapy

**DOI:** 10.4103/0971-6203.29197

**Published:** 2006

**Authors:** P. Reena, S. Dayananda, Rajeshri Pai, S. V. Jamema, Tejpal Gupta, D. Deepak, S. Rajeev

**Affiliations:** Department of Radiation Oncology, ACTREC, TMC, Kharghar, New Mumbai, India; * Department of Medical Physics, Tata Memorial Hospital, TMC, Parel, Mumbai, India

**Keywords:** Performance characterization, small monitor unit, small segment, step-and-shoot intensity-modulated radiotherapy

## Abstract

Implementation of step-and-shoot intensity-modulated radiotherapy (IMRT) needs careful understanding of the accelerator start-up characteristic to ensure accurate and precise delivery of radiation dose to patient. The dosimetric characteristic of a Siemens Primus linear accelerator (LA) which delivers 6 and 18 MV x-rays at the dose rate of 300 and 500 monitor unit (MU) per minutes (min) respectively was studied under the condition of small MU ranging from 1 to 100. Dose monitor linearity was studied at different dose calibration parameter (D1_C0) by measuring ionization at 10 cm depth in a solid water phantom using a 0.6 cc ionization chamber. Monitor unit stability was studied from different intensity modulated (IM) groups comprising various combinations of MU per field and number of fields. Stability of beam flatness and symmetry was investigated under normal and IMRT mode for 20×20 cm^2^ field under small MU using a 2D Profiler kept isocentrically at 5 cm depth. Inter segment response was investigated form 1 to 10 MU by measuring the dose per MU from various IM groups, each consisting of four segments with inter-segment separation of 2 cm.

In the range 1-4 MU, the dose linearity error was more than 5% (max −32% at 1 MU) for 6 MV x-rays at factory calibrated D1_C0 value of 6000. The dose linearity error was reduced to −10.95% at 1 MU, within −3% for 2 and 3 MU and ±1% for MU ≥4 when the D1_C0 was subsequently tuned at 4500. For 18 MV x-rays, the dose linearity error at factory calibrated D1_C0 value of 4400 was within ±1% for MU ≥3 with maximum of −13.5 observed at 1 MU. For both the beam energies and MU/field ≥4, the stability of monitor unit tested for different IM groups was within ±1% of the dose from the normal treatment field. This variation increases to −2.6% for 6 MV and −2.7% for 18 MV x-rays for 2 MU/field. No significant variation was observed in the stability of beam profile measured from normal and IMRT mode. The beam flatness was within 3% for 6 MV x-rays and more than 3% (Max 3.5%) for 18 MV x-rays at lesser irradiation time ≤3 MU. The beam stability improves with the increase in irradiation time. Both the beam energies show very good symmetry (≤2%) at all irradiation time.

For all the three segment sizes studied, the nonlinearity was observed at smaller MU/segment in both the energies. When the MU/segment is ≥4, all segment size shows fairly linear relation with dose/MU. The smaller segment size shows larger nonlinearity at smaller MU/segment and become more linear at larger MU/segment. Based on our study, we conclude that the Primus LA from Siemens installed at our hospital is ideally suited for step-and-shoot IMRT preferably for radiation ON time ≥4MU per segment.

Intensity-modulated radiotherapy (IMRT) is increasingly adopted in the treatment of several types of cancer because of its superior dose conformity to irregular concave shaped target volume and conformal avoidance of nearby critical organs. In this technique, intensity modulated beam (IMB) generated using inverse planning strategies and optimization algorithms on the treatment planning system (TPS) is delivered through varied means in the linear accelerator (LA).[1–3] In step-and-shoot IMRT, each IMB is delivered through a series of complex small segmented fields employing small monitor unit (MU). Some of these segments may be smaller than 3×3 cm^2^ and may deliver as small as 2-3 MU at different off-axis distances. Generally, the dosimetric performance of the LA is evaluated under normal treatment condition employing large field sizes greater than 3×3 cm^2^ and MU more than 50. Therefore, above the acceptance testing of LA for normal treatment, implementation of IMRT needs extensive pre-commissioning performance characterization of all systems involved.[1,4,5] Accurate and precise delivery of planned dose to the tumor from step-and-shoot IMRT will mainly depend on the performance of the LA under small segments of radiation delivery with small MU. In this work, performances of a newly installed Siemens Primus LA were evaluated for dose monitor linearity, monitor unit stability, stability of beam flatness and symmetry and inter segment response for the implementation of step-and-shoot IMRT.

## Materials and Methods

A dual energy standing wave accelerator (Siemens Primus, Siemens Medical Systems, Concord, CA, USA) equipped with 29 pairs of double-focused MLC and Primeview was commissioned for IMRT treatment delivery. The projected leaf width at isocenter for the central 27 leaf pairs is of 1 cm while the peripheral two pairs are of 6.5 cm. Each leaf can travel a distance of 10 cm beyond the central axis and tongue and groove mechanism was used to minimize interleaf leakage. The detailed performance characteristics of this MLC for step-and-shoot IMRT have been described elsewhere.[6]

Siemens Primus LA used in this study can deliver x-rays of nominal energies 6 and 18 MV operated at a dose rate of 300 MU/min and 500 MU/min, respectively. During the step-and-shoot delivery of IMRT, radiation is turned off by desynchronizing the injector while the field parameters are being changed. When the machine is ready again a trigger pulse is send to the injector to start the beam instantaneously. The Primeview (Version 2.1.659), a Siemens software interface to the LA enable the creation of intensity modulated (IM) group for each treatment field and delivery of an entire treatment in auto sequencing mode to simulate IMRT delivery.

### Dose monitor linearity

Linearity of MU was studied within the range of 1-100 MU by measuring the corresponding dose from 6 and 18 MV X-rays using 10×10 cm^2^ field size. For 6 MV x-rays, all the measurements were performed at dose calibration parameter (D1_C0) set at 6000 using a 0.6 cc ionization chamber connected to a calibrated electrometer (Dose 1, Scanditronix Wellhofer, Sweden) and kept at 10 cm depth in a solid water phantom (Standard Imaging, Middleton, WI, USA) under isocentric condition. Five sets of ionization readings were recorded for each MUs ranging from 1 to 10 MU in increments of 1 MU and for every 10 MU thereafter up to 100 MU. A normalization factor (NF) was calculated by taking the ratio of 100 MU to the corresponding average dose. The normalized averages (NA) were calculated for each MU setting by multiplying the corresponding average doses by the normalization factor (NF). The dose linearity error (δ) was then calculated for each set MU using the relation; δ = [NA/MU programmed - 1]×100. The same measurements were repeated for D1_C0 set at 4500. Similar measurements were repeated for 18 MV X-rays and D1_C0 set at 4400.

### Monitor unit stability

To test the stability of MU in the dose rate of 300 MU/min for 6 MV x-rays and 500 MU/min for 18 MV x-rays, intensity modulated (IM) group was prepared in Primeview by arranging multiple 10×10 cm^2^ fields having same MU per field in auto-sequence mode. This simulates step-and-shoot delivery technique wherein the LA is temporarily in the ‘Pause Mode’ after delivering the dose from the first field and turn ‘ON’ again once the MLC take the second field shape. First a total of 100 MU was delivered from a 10×10 cm^2^ field and the measured dose represents the reference dose in normal treatment mode. Then the dose from the same 100 MU was measured in IMRT mode using 5 fields of 10×10 cm^2^ in auto-sequence, each field delivering 20 MU. Similar measurements were made with 10 MU/field and 10 fields, 5 MU/field and 20 fields, 4 MU/field and 25 fields and 2 MU/field and 50 fields in auto-sequence. Variation of measured dose from these different combinations of MU/field and number of fields was calculated with respect to the reference dose. In this experiment, the same measurement condition for dose monitor linearity was used.

### Stability of beam flatness and symmetry

Beam flatness and symmetry under accelerator start up condition (1-10 MU) was studied by measuring in-line and cross-line profiles from a 20×20 cm^2^ field using a 2D Profiler (Profiler Model 1170, Sun Nuclear Corporation, Melbourne, FL) kept isocentrically at 5 cm depth. The 2D Profiler consists of 46 diodes, with the adjacent diodes separated by a distance of 0.5 cm and it acquires beam profile data at a rate of ten frames per second. These profiles were compared with profiles acquired under the same geometry with larger MUs of the order of 50-100 MU. Intensity modulated (IM) group consisting of 10 fields each of 20×20 cm^2^ and having different MUs ranging from 1 to 10 in increment of 1 MU and another two fields with 50 and 100 MU were made in the Primeview. The beam profiles from these fields were measured in the IM mode using the same profiler and set-up geometry.

### Inter-segment variations

IM group consisting of four segments, each of 1×10 cm^2^ with inter-segment separation of 2 cm were made in the Primeview. Another two IM groups were made in a similar manner using segment size of 1.5×10 and 2×10 cm^2^, respectively. Dose from these IM groups were measured at 10 cm depth for different MU per segment settings of 1,2,3,5,7 and 10, respectively. Inter segment response was investigated from the dose per MU estimated from these three IMB. All the above measurements were made on a solid water phantom (Standard Imaging, Middleton, WI, USA) using a 0.6 cc ionization chamber (Dose 1, Scanditronix Wellhofer, Sweden).

## Result

### Dose monitor linearity

The dose linearity error within the range of 1-100 MU measured at different dose calibration parameter (D1_C0) for 6 MV x-rays and factory calibrated D1_C0 for 18 MV x-rays are summarized in Table 1. In the range 1-4 MU, the dose linearity error was more than 5% (max −32% at 1 MU) for 6 MV x-rays at factory calibrated D1_C0 value of 6000. The dose linearity error was reduced to within −3% for 2 and 3 MU with maximum of −10.95% at 1 MU when the D1_C0 subsequently tuned at 4500. This adjustment in D1_C0 value also leads to overall improvement in dose linearity. When D1_C0 is set at 4500, MU ≥4 gives dose linearity error less than ±1%. However for 18 MV x-rays, the dose linearity error at factory calibrated D1_C0 value of 4400 was within ±1% for MU ≥3 with maximum of −13.5 observed at 1 MU. All subsequent measurements were carried out at D1_C0 set at 4500 for 6 MV and 4400 for 18 MV x-rays.

**Table 1:** Dose monitor linearity error for 6 and 18 MV X-ray at different monitor unit ranges and dose calibration parameters D1_C0

*Set*	*Dose linearity error %*		
	
*MU*	*6 MV X-ray*		*18 MV X-ray*

	D1_C0=6000	D1_C0=4500	D1_C0=4400
1	−32.00	−10.95	−13.50
2	−12.90	−2.42	−1.55
3	−8.12	−2.28	−0.93
4	−6.20	−0.53	0.72
5	−2.77	0.70	−0.68
6	−2.50	−0.85	0.90
7	−2.30	−0.48	−0.41
8	−2.10	−0.54	0.19
9	−1.90	−0.29	0.90
10	−1.75	−0.51	0.50
20	−1.10	0.00	−0.20
30	−0.80	0.07	−0.30
40	−0.50	0.00	−0.26
50	0.20	0.05	0.17
60	0.30	0.04	−0.09
70	0.40	0.16	−0.04
80	0.10	0.21	−0.13
90	0.15	0.24	0.02
100	0.00	0.00	0.00

### Monitor unit stability

The dose per MU measured from various IM groups represented by the different combination of MU/field and number of fields was compared against the dose measured from the normal treatment field of 10×10 cm^2^ having same 100 MU [Table 2]. For both the beam energies and MU/field ≥4, the stability of monitor unit tested for different IM groups was found satisfactory and within ±1% of the dose from the normal treatment field. This variation was found to increase for 2 MU/field, measuring −2.6% for 6 MV and −2.7 % for 18 MV x-rays.

**Table 2:** Stability of monitor unit (MU) at normal and intensity modulated mode having different MU/fields and number of fields

*MU/field*	*No. of field (s)*	*Dose (cGy) per MU %*		*Variation from reference dose*	
			
		*6 MV*	*18 MV*	*6 MV*	*18 MV*
100	1	0.80 (ref)	0.930 (ref)	0	0
20	5	0.801	0.931	+0.13	+0.11
10	10	0.799	0.929	−0.13	−0.11
5	20	0.799	0.929	−0.13	−0.11
4	25	0.798	0.927	−0.25	−0.32
2	50	0.779	0.905	−2.6	−2.7

MU - Monitor unit

### Stability of beam flatness and symmetry

The composite beam profile at small (1 MU) and large (100 MU) irradiation time under normal and IM mode are shown in Figure 1a and 1b for 6 MV X-rays and Figure 2a and 2b for 18 MV x-rays, respectively. For both the beam energies the deviations between the beam flatness and symmetry measured at low (<10 MU) and large irradiation time (>10 MU) were found to be insignificant in both normal and IMRT mode. Table 3 summarizes the flatness and symmetry at different MU ranging from 1 to 100 for both the beam energies and irradiation mode. For 6 MV x-rays, beam flatness along cross plane (X) and in-plane (Y) was found to be within 3%. Whereas, the beam flatness for 18 MV x-rays was more than 3% (Max. 3.5%) at lesser irradiation time ≤3 MU. Both the beam energies show very good symmetry (≤2%) at all irradiation time and irradiation mode.

**Figure 1a:**
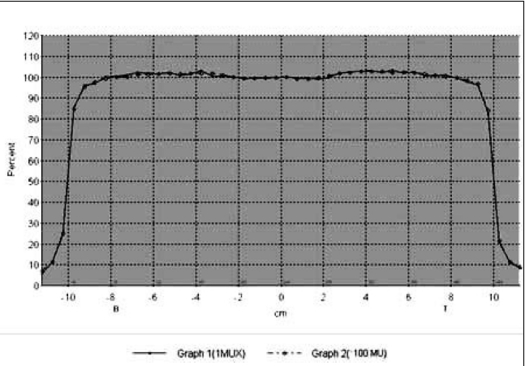
Composite cross profile of 1 MU and 100 MU for 6 MV X-ray under normal mode

**Figure 1b:**
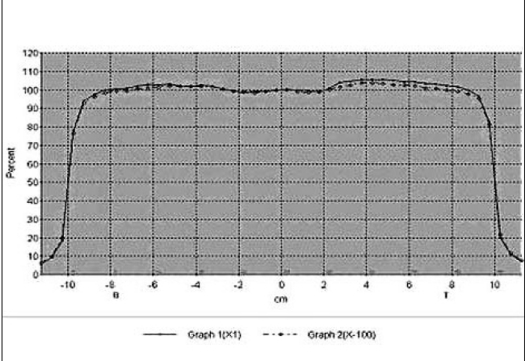
Composite cross profile of 1 MU and 100 MU for 6 MV X-ray under IM mode

**Figure 2a:**
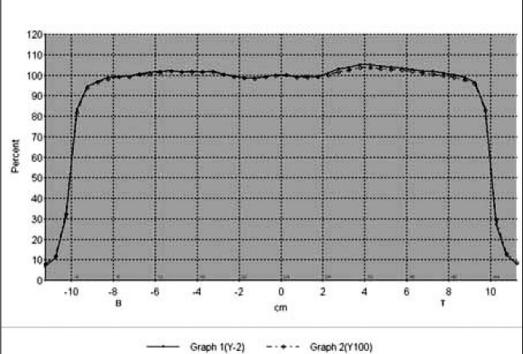
Composite In-line profile of 1MU and 100 MU for 18 MV X-ray under normal mode

**Figure 2b:**
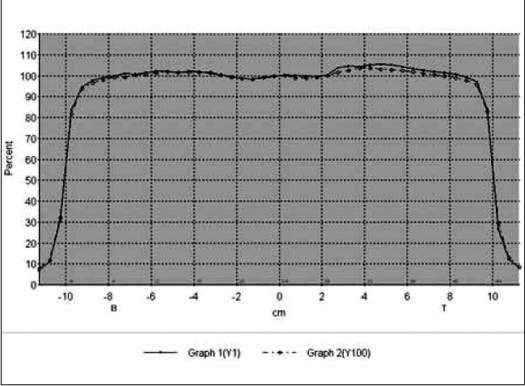
Composite In-line profile of 1MU and 100 MU for 18 MV X-ray under IM mode

**Table 3a:** Flatness and symmetry value for 6 MV X-ray at different monitor unit in normal mode and intensity modulated mode

*Set MU*	*Normal mode*				*IM mode*			
		
	*Cross-line*		*In-line*		*Cross-line*		*In-line*	
				
	*Flatness (%)*	*Symmetry (%)*	*Flatness (%)*	*Symmetry (%)*	*Flatness (%)*	*Symmetry (%)*	*Flatness (%)*	*Symmetry (%)*
1	2.1	0.4	2.5	1.2	2.6	0.7	2.7	1.2
2	2.1	0.4	2.1	0.9	2.4	0.7	2.6	1.2
3	1.9	0.4	2.2	0.6	2.4	0.5	2.6	1.0
4	1.9	0.5	2.1	0.7	2.1	0.6	2.3	0.9
5	1.9	0.5	2.2	0.6	2.1	0.5	2.3	1.0
6	1.9	0.5	2.1	0.6	2.1	0.6	2.1	0.8
7	1.8	0.4	2.1	0.6	2.5	0.5	2.2	0.8
8	1.8	0.5	1.9	0.5	1.9	0.4	2.1	0.6
9	1.8	0.5	2.0	0.6	2.0	0.4	1.8	0.6
10	1.8	0.5	2.0	0.5	1.9	0.5	1.8	0.6
50	1.7	0.5	2.0	0.5	1.8	0.4	2.0	0.7
100	1.8	0.4	2.0	0.5	1.8	0.4	1.8	0.6

MU - Monitor unit, IM - Intensity modulated

**Table 3b:** Flatness and symmetry value for 18 MV X-ray at different monitor unit in normal mode and intensity modulated mode

*Set MU*	*Normal mode*				*IM mode*			
		
	*Cross-line*		*In-line*		*Cross-line*		*In-line*	
				
	*Flatness (%)*	*Symmetry (%)*	*Flatness (%)*	*Symmetry (%)*	*Flatness (%)*	*Symmetry (%)*	*Flatness (%)*	*Symmetry (%)*
1	3.4	1.9	3.5	2.0	3.3	2.0	3.5	2.0
2	3.1	1.5	3.2	1.7	2.9	1.7	3.3	1.8
3	3.2	1.3	3.2	1.6	3.0	1.5	2.9	1.7
4	3.0	1.2	3.0	1.6	2.9	1.5	3.0	1.4
5	3.0	1.3	2.8	1.4	3.1	1.4	3.1	1.5
6	3.0	1.3	2.9	1.4	3.0	1.5	2.9	1.4
7	2.9	1.1	3.0	1.5	3.0	1.2	2.9	1.6
8	3.0	1.3	3.0	1.6	2.8	1.1	2.9	1.5
9	2.9	1.2	3.0	1.6	2.9	1.3	3.0	1.5
10	3.0	1.3	3.0	1.6	2.8	0.9	2.8	1.3
50	2.7	0.9	3.0	1.4	2.7	0.7	2.8	1.2
100	2.7	0.8	2.6	0.9	2.8	0.8	2.5	1.1

MU - Monitor unit, IM - Intensity modulated

### Intersegment variations

**Figure 3:**
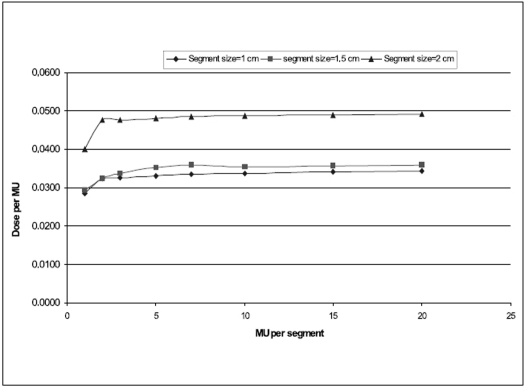
Variation of dose/MU with MU/segments for 6 MV X-rays

**Figure 4:**
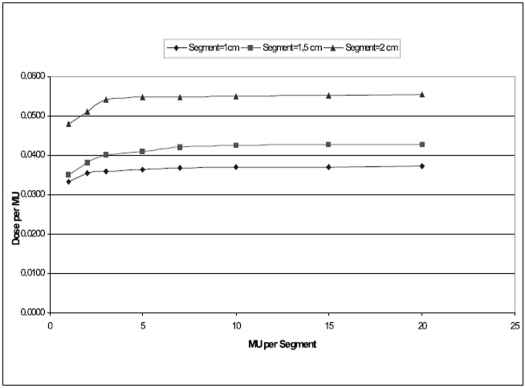
Variation of dose/MU with MU/segments for 18 MV X-rays

The variation of dose/MU with respect to set MU/segment for different segment sizes are shown in Figure 3 for 6 MV X-rays and [Figure 4] for 18 MV X-rays. For all the three segment sizes studied, the nonlinearity was observed at smaller MU/segment in both the energies. When the MU/segment is ≥4, all segment size shows fairly linear relation with dose/MU.

## Discussion

Several authors have investigated accelerator startup (small MU) characteristics under normal treatment condition.[7–9] It has been recommended that when low doses are required proper precautions should be taken for dosimetric accuracy including the beam energy, beam flatness and dose per monitor unit.[7–9] Sharpe *et al.*[10] have reported the MU linearity, beam flatness and symmetry for step-and-shoot IMRT delivery using a traveling wave linear accelerator. Cheng *et al*.[11] have compared the beam start up characteristics of a standing wave accelerator (Siemens Primus) for normal and step-and-shoot IMRT delivery.

The dose monitor linearity for 6 MV x-rays at the dose rate of 300 MU/min and factory calibrated D1_C0 value of 6000 was higher than the manufacturer specified value of ±3% for MU range of 1-10. This led us to the adjustment of D1_C0 value. In Siemens LA, D1_C0 represents a dosimetry offset applied to the dose monitor 1 (D1) gain. It allows the MU counter to start and terminate earlier with the intension of minimizing monitor-end errors. At the optimum D1_C0 set at 4500, the dose linearity was achieved within ±3% except for 1 MU. The large nonlinearity observed at very small (1 MU) irradiation time may be due to partly the inaccuracy associated with low dose measurement and monitor start or end error. The estimated uncertainties of the measurement were ±2% for 1-3 MU, ±1% for 4-10 MU and less than 1% for MU greater than 10. Above 4 MU dose monitor linearity was well within the manufacturer specified limit of ±2 and ±1% for 11-20 and 21-100 MU, respectively. For 18 MV X-rays operated at the dose rate of 500 MU/min and factory calibrated D1_C0 value of 4400, the dose was linear with MU within the manufacturer specified limit of ±3, ±2 and ±1% for MU ranges from 2-10, 11-20 and 21-100, respectively. However, similar to the finding in 300 MU/min dose rate, the dose monitor linearity for 1 MU was more than the specified limit of ±10%. Our findings are similar to the values reported by others.[5] All the other measurements were carried out at D1_C0 set at 4500 for 6 MV and 4400 for 18 MV x-rays. For both the beam energies and MU/field ≥2, the monitor unit was stable within the manufacturer specified limit of ±3%. The stability of beam profile in normal and IMRT modes was within the manufacturer specified limit for 6 MV X-rays. For 18 MV x-rays operated at 500 MU/min, the beam stability is slightly reduced in the MU range lesser than 3. This may affect the precision of the relative output when beams are directed asymmetrically off-axis.

## Conclusion

The dosimetric study of the Siemens Primus LA shows that beam uniformity, symmetry and dose linearity were independent of MU and treatment mode for treatment time greater than 1 MU. Inter segment MU deliveries are almost linear for all beam segment independent of segment width and MU setting when MU/segment is ≥4. Based on our study, we conclude that the Primus LA from Siemens installed at our hospital is ideally suited for step-and-shoot IMRT preferably for radiation ON time ≥4 MU per segment.
